# Comparative mechanical analysis of deep brain stimulation electrodes

**DOI:** 10.1186/s12938-018-0557-6

**Published:** 2018-09-18

**Authors:** H. H. Draz, S. R. I. Gabran, Mohamed Basha, Hassan Mostafa, Mohamed F. Abu-Elyazeed, Amal Zaki

**Affiliations:** 1 Department of Microelectronics, Electronics Research Institute, El Tahrir st, El Dokki, 12622 Giza, Egypt; 2 Electronics and Communications Department, Faculty of Engineering, Cairo University, 12316 Giza, Egypt; 3 Department of Electrical and Computer Engineering with the Center for Integrated RF Engineering (CIRFE Lab), University of Waterloo, Waterloo, Canada; 4 Department of Electrical and Computer Engineering with the Centre for Intelligent Antenna and Radio System (CIARS), University of Waterloo, Waterloo, Canada; 5 Nanotechnology and Nanoelectronics Devices Program, Zewail City of Science and Technology, 6th of October, Egypt

**Keywords:** Microelectrode, Finite element model, Buckling analysis, Stationary analysis, FOM

## Abstract

The new field of neuro-prosthetics focuses on the design and implementation of neural prostheses to restore some of the lost neural functions. The electrode-tissue contacts remain one of the major obstacles of neural prostheses microstructure. Recently, Microelectrode fabrication techniques have been developed to have a long-term and stable interface with the brain. In this paper, a comparative analysis of finite element models (FEM) for several electrode layouts is conducted. FEM involves parametric and sensitivity analysis to show the effects of the different design parameters on the electrode mechanical performance. These parameters include electrode dimensions, geometry, and materials. The electrodes mechanical performance is evaluated with various analysis techniques including: linear buckling analysis, stationary analysis with axial and shear loading, and failure analysis for brittle and ductile materials. Finally, a novel figure of merit (FOM) is presented and dedicated to the various electrodes prototypes. The proposed designs take into account mechanical performance, fabrication cost, and cross sectional area of the electrode. The FOM provides important design insights to help the electrodes designers to select the best electrode design parameters that meet their design constraints.

## Background

Recently deep brain stimulation microelectrode has become an essential component in the therapy of neural disorders. The chronical electrodes are implanted into the selected brain target and are directed to treat the movement disorder. During the past decades, deep brain electrodes have been developed to study the functions of the nervous system. Glass micropipettes were the first form of microelectrodes but are limited by single site in vitro recording for research applications [1]. Glass micropipettes were replaced by microwire bundle electrodes in rodent and primate signal recording. Microwires were the first implantable electrodes. They were used to record chronically from the brain to focus on the individual neuron, and offered multiple recording sites [2, 3]. Microwire was one of the three common fabrication techniques of implemented microelectrodes [2]. The disadvantages of microwire electrodes are: (1) limited to single channel per wire, and (2) during implantation, the accurate location of the electrode tips relative to each other is not controllable due to wire bending. A number of innovations along with the advancement of micro-fabrication technology leads to the advent of micromachined electrodes, which are considered the second type of fabrication techniques. Micromachined electrodes overcome the drawbacks of microwire electrodes by using a rigid structure of Silicon, or a metal-based array implemented by electrical discharge machining (EDM) technique associated with electrochemical steps [4]. The Silicon electrode has two models: (1) patterning the electrode sites on the shank of Silicon substrate (2) isolated sharpened Silicon needles. It provides a high density of sensors with predetermined locations and high spatial resolution.

The third type of fabrication techniques of implemented microelectrode is the flexible electrodes [2]. The flexible electrodes achieve an advantage over rigid electrodes because of the matching between brain tissue and the nature of these electrodes. Microelectrode fabrication technique assists in getting consistent recording signals without losing micro-stimulation capabilities from small group of neurons.

The materials of the electrodes and geometries are altered to maintain low impedance path for charge injection, high charge transfer, and high spatial resolution. A small electrode size is required, but this increases impedance and affects the signal to noise ratio (SNR). The electrode fabrication design parameters such as shape, materials, and fabrication technique should be optimized to achieve the best electrode performance. The mechanical performance of the electrode is affected by three main parameters, which are; the layout, the material and the geometry of this electrode [5]. The electrode design focal issue is to minimize the tissue trauma. In addition, the electrode should have a rigid structure to be capable of penetrating through the tissue without the need of any other assistive devices for successful insertion. The structure of the electrode should be flexible to match the tissue stiffness and to minimize post implementation tissue trauma. The challenges facing the electrode design are the electrode flexibility and rigidity. To improve the electrode biocompatibility, the mechanical properties should be provided through a small footprint design. Several layouts are patterned and analyzed in order to identify the qualified designs, satisfying all the mechanical requirements. These layouts are analyzed using COMSOL multiphysics.

The estimated insertion force and the mechanical failure modes are identified for brittle and ductile materials [1, 6]. A comparison between several electrode layouts is presented to study which design achieves the best critical load and safety factor with respect to axial loading and shear forces. The main degree of freedom is the shank thickness for a constant shank width. The minimum value of the shank width depends on the minimum dimensions of the stimulation pads and this is why the shank width is considered to be constant [6]. The critical load and the safety factors of the electrode are calculated for several layouts with different materials. Finally, strain relief sections are modeled to improve the electrode mechanical performance and enhance its biocompatibility.

Some modifications are adopted to reinforce the microelectrode to have a long-term and stable interface with the brain.

Finally, a FOM is calculated for each design, which is considered as a numerical quantity based on one or more characteristics of the design to represent a measure of efficiency. Low impedance, low noise, low fabrication cost, small cross section area, large number of channels, and high safety factor of mechanical analysis are the characteristics of FOM [7].

In this work, the comparative analysis is among the designs with different fabrication cost, different dimensions, different safety factors, and keeping the other characteristics fixed for all designs.

## Methods

### Electrode forces and failure models

#### Forces acting on the electrode

**Fig. 1:**
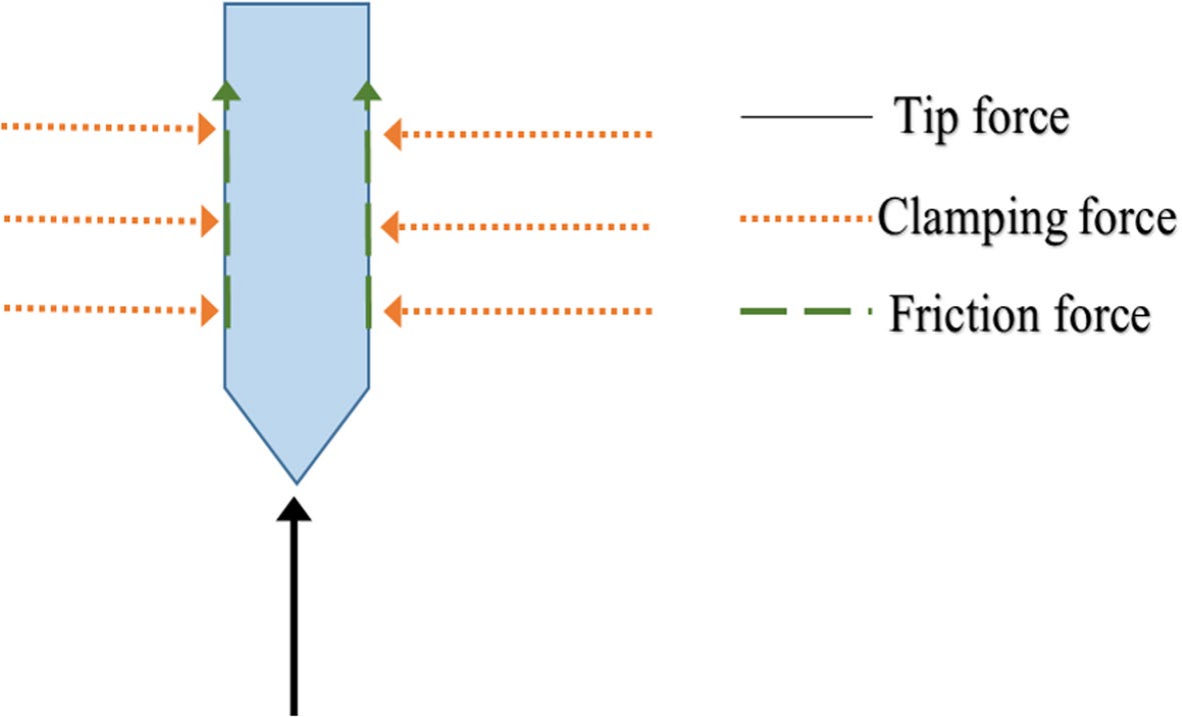
Forces affecting the electrode. there are three mechanical forces acting on the electrode, these forces are outlined on this free body diagram

During insertion, there are three mechanical forces acting on the electrode, these forces are outlined on the free body diagram in Fig. 1 [8]. The tip force is the axial reaction acting on the electrode tip during penetration. The clamping force is the force normal to the electrode surface. During insertion, the normal force acting on the surface and the coefficient of friction produce friction force along the electrode surface. The total axial reaction force acting on the electrode is calculated from these forces. The applied insertion force should be greater than the total reaction force to achieve successful penetration without any of mechanical failures.

A wide range of penetration forces are defined from the mechanical perspective of various types of tissues and estimated to be from 1 to 10 mN [9]. This is lower than the penetration force of epidermal tissue by 2–3 orders of magnitude. This epidermal tissue penetration force is estimated to be 1 N [3].

#### Modes of mechanical failure

During insertion, the electrode is subjected to two mechanical failure modes: buckling and fracture [10]. The electrode structural layer is tested with several materials, and different geometries. Each material has different failure modes depending on the material mechanical properties.

Two types of materials are used in the implementation of the electrodes: Brittle materials (e.g. Silicon) and ductile materials (e.g. metals and flexible polymers). Metals as Copper and Nickel are implemented as a substrate and coated with thin layer of gold to be biocompatible with tissues.

Under uniaxial stresses testing, the Brittle materials suffer from elastic deformation until the stress level exceeds the yield strength then, they show negligible plastic deformation followed by fracture. Therefore, the maximum loading limit for brittle structures is considered as the design parameter ultimate tensile strength (UTS). On the other side, when applying uniaxial stress on the ductile materials, they suffer from elastic deformation until reaching the yield point, followed by plastic deformation and finally cracking leading to fracture. When the maximum shear stress exceeds the maximum shear yield strength of the material, ductile materials fail. Some of properties of the used materials are illustrated in Table 1.

**Table 1:** Materials properties

Material	Young’s modulus (GPa)	Poison ratio	Density (Mg m^−3^)	Ultimate tensile stress (MPa)	Shear modulus (GPa)	Yield stress (MPa)
Copper alloys	135	0.35	8.3	720	50	510
Nickel alloys	180	0.31	8.5	1200	70	900
Polyamide (nylon)	3	0.42	1.1	55	0.76	40
Silicon	185	0.28	2.33	35	79.9	7000

### Electrode prototypes and mechanical analysis

#### Electrode prototypes

**Fig. 2:**
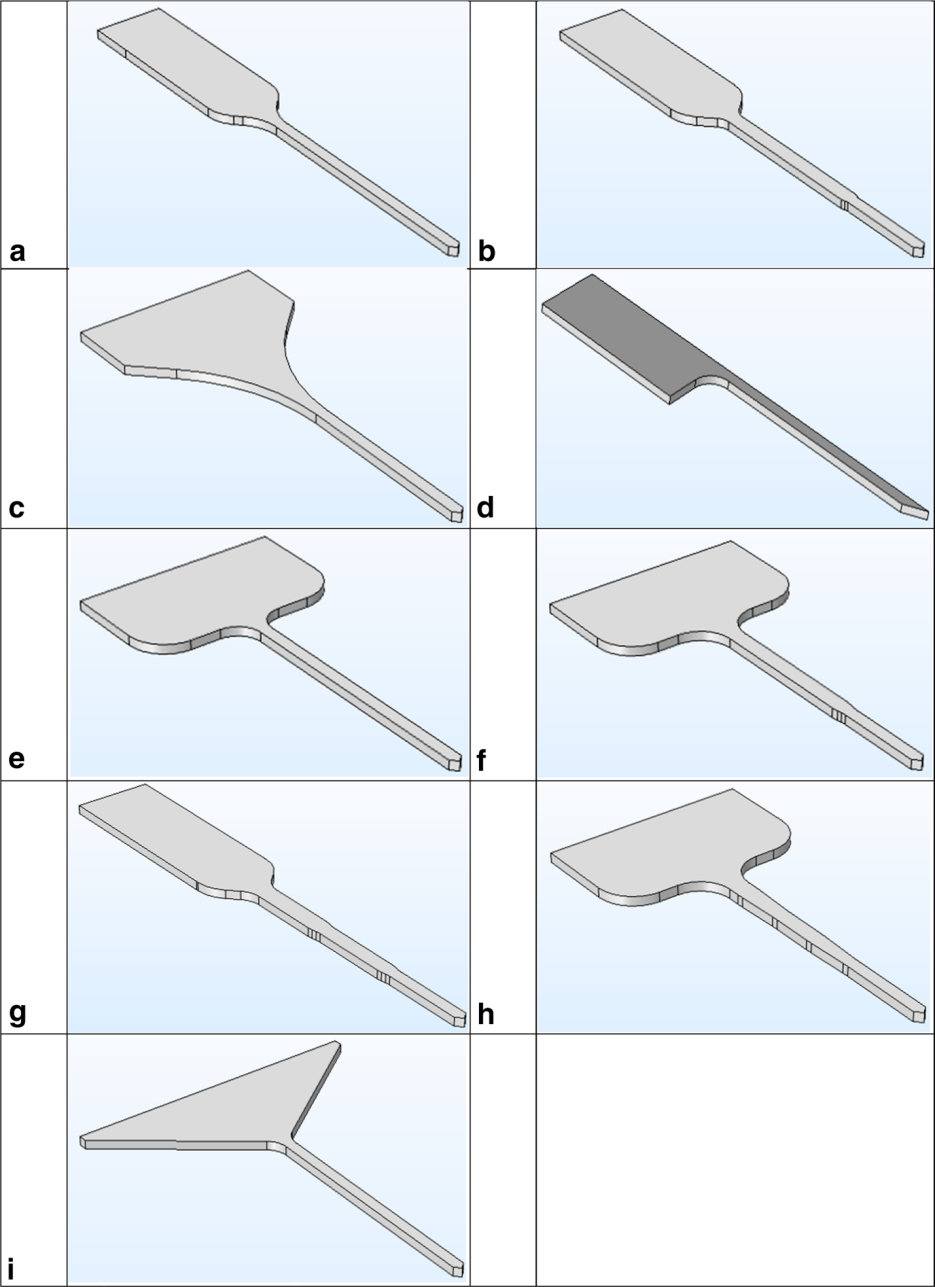
Several layout designs of the electrodes labled as Prototype** a**,** b**,** c**,** d**,** e**,** f**,** g**,** h** and** i**

Several electrode layouts are introduced and their mechanical properties are tested in the pursuit of a design that accommodates a large number of stimulation channels. The proposed layouts are presented in Fig. 2. In general, the bases are designed with big size to facilitate treatment and provide large interconnect pads for easy connections, while the length of the prototypes shanks is enough to reside within 3 mm inside the tissue and carry the interface pads.

The interconnect pads on prototypes C, E, F, H and I are arranged transversely, while those in A, B, D and G are longitudinal. The simulations of the electrode models are analyzed to explore their mechanical performance as well as the effect of the electrode material and dimensions on the critical load. In addition, parametric modeling and sensitivity analysis are employed.

#### Electrode mechanical analysis

*Buckling analysis* Buckling is a failure mode that appears in thin structures when the axial force applied on the shank exceeds the critical load; which is known as the threshold value, and disturbs its structural equilibrium. This leads the shaft into an unstable state and the electrode shank geometry falls into buckling modes [6].

During electrode insertion, the instance just before tissue penetration is the most critical, where the electrode effective length and the axial load are at their maximum values [11]. The value of the critical load depends on the material, the electrode geometry and the end supports configuration.

Buckling resistance decreases with the presence of geometrical asymmetry, material defects, and eccentric loading which introduces bending moments and promotes curvature. The electrode should be designed to have a critical load higher than the force required for tissue penetration; to avoid buckling failure, otherwise; an insertion support tool is required. The electrode is modeled for linear buckling analysis and the different layouts are also modeled and analyzed. The critical loads are estimated to represent the forces that excite the buckling failure. While the tip is constrained in all directions to create fixed-free loading condition, unity axial force is applied to the electrode base. Eventually the buckling mode is extracted and the critical load is estimated [9], and then safety factor is calculated according to Eq. (1) [12]

1 $$\begin{aligned} \text {Safety factor} = & {} \frac{\text {failure load}}{\text {design load}} \end{aligned}$$

where: the Failure load is the Critical load.

Design load: penetration force estimated to be 1 mN [13].

To investigate the effect of different electrode parameters on its mechanical performance, a shank with uniform rectangular cross-section is modeled. The thickness is varied from 20 to 200 μm while the shank width is fixed at 130 μm. A 3 mm Silicon or Nickel shank with thickness of 30 μm has a critical load of  5 mN, which dropped to  0.1 mN for Polyimide shank. Polyimide sheet, Copper, Silicon, and Nickel photo-resist are modeled as the structural material. To survive a critical load of 10 mN with Polyimide rectangular shank, a thickness varying from 120 to 180 μm is required depending on the electrode geometry.

*Fracture* The electrode is subjected to lateral forces normal to the shank surface, during implantation and operation. These forces induce shear stresses [14]. Two sets of analysis are executed and fixed free support conditions are assumed.

For ductile materials, the maximum von mises stress is assumed to quantify the electrode failure. The electrode model is configured for stationary analysis and the loading force value is set to the value of insertion force. The von mises stress is the result of the stationary analysis, illustrates the magnitude of stress levels and identifies high stress regions. For the analysis of brittle materials, the maximum principal stress theory (Rankine criterion) is adopted. It states that failure occurs when the maximum principal stress, determined from uniaxial loading, reaches the ultimate tensile strength (UTS), and similarly for compressive stress. The three principal stresses at any point ($\sigma _1,\sigma _2,\sigma _3$) might include tensile and compressive components. The smallest negative value represents the maximum compressive stress, while the largest positive value represents the maximum tensile stress. This is due to the fact that brittle materials fail by fracture upon reaching the ultimate tensile stress, but do not yield nor endure plastic deformation [9].

COMSOL stationary analysis tool is utilized for calculating the fracture safety factor using suitable criteria with respect to material properties. The minimum safety is considered a safety factor of 5 [6]. In general, brittle materials fail in shear, while ductile materials fail in axial. For ductile materials, compression strength and tension are roughly equal, however, compression strength is much greater than tensile strength in brittle materials. The safety factor for axial loading is calculated by comparing the yield strength and von mises stress using Eq. 2 [12]. The accepted value is greater than or equal to 5. Finally, safety factor for shear force is calculated as the ratio between the maximum shear strength and the maximum shear stress of the design as illustrated in Eq. 3. The maximum shear stress of the design depends on the maximum principle stress ($\sigma _1,\sigma _3$) as illustrated in Eq. 4.

2 $$\begin{aligned} \text {Safety factor with axial load} = & {} \frac{\text {yield stress}}{\text {von miss stress}} \end{aligned}$$

3 $$\begin{aligned} \text {Safety factor with shear stress} = & {} \frac{\text {max. shear strength}}{\text {max. shear stress in design}} \end{aligned}$$

4 $$\begin{aligned} \text {max. shear stress} = & {} \frac{\sigma _1-\sigma _3}{2} \end{aligned}$$

## Fabrication process and costs

The fabrication steps and costs have been estimated for each material. It should be known that some steps might need further development in order to get the electrode properly with its required structure. The main fabrication steps implemented with copper and nickel are as follows:

- RCA-1 Si wafer cleaning.
- Sputtering of thin layer of metal (Cu, Ni).
- Lithography and spin coating of more than 100 μm thick Photoresist.
- Cu electroplating.
- Performing chemical mechanical polishing CMP to improve surface roughness.
- RCA-1 cleaning and removing photoresist.
- Gold plating to make the structure bio-compatible, since Ni and Cu are poisonous materials.

However, Nickel is a high stress material, thus curvatures may be encountered during the structure release. As for Si material, SOI wafers will be used instead of Si wafers, thus there is no need for sputtering step. The fabrication steps are as shown:

- RCA-1 SOI wafer cleaning.
- Lithography and spin-coating of photoresist.
- Deep reactive ion etching (DRIE) of the wafer.
- RCA-1 SOI wafer cleaning.
- Dicing of the SOI wafers to get individual electrodes.
- Back side etching of the Si handle wafer to release the structure using KOH.
- HF to remove oxide layer in the SOI wafer.

As for the Polyimide material, simpler steps are needed. The main step is lithography in order to achieve the required thickness and using the appropriate developer.

## Results

### Linear buckling

Thin Silicon and Nickel structures satisfy the mechanical requirements and avoid buckling failure. They score a minimum safety factor of 23 for layout A with thickness of 50 μm. On the other hand, the minimum thickness of a Polyimide electrode, to barely survive buckling, is 100 μm with a minimum safety factor of 3.1.

Table 2, illustrates the safety factor of each layout with different materials and different thicknesses. Demonstrated results show that layouts E, F and H are the most stable, while layout A is the most vulnerable to buckling failure because it has the longest base length.

**Table 2:** Safety factor for different layouts: fixed-free linear buckling analysis

	Si			Cu			Ni			Polyimide		
	20 μm	100 μm	200 μm	20 μm	100 μm	200 μm	20 μm	100 μm	200 μm	20 μm	100 μm	200 μm
A	1.48	185.38	652.47	0.96	120.4	423.67	1.76	219.57	772.76	0.0249	3.11	10.95
B	1.71	213.72	934.36	1.11	138.83	607.09	2.03	253.16	1106.95	0.0287	3.59	15.68
C	2	251.36	899.21	1.30	163.35	584.03	2.37	297.81	1065.1	0.0336	4.22	15.09
D	1.75	216.84	761.38	1.23	153.1	538.01	2.25	279.37	981.32	0.0319	3.96	13.9
E	1.66	274.96	943.87	1.56	194.39	667.03	2.84	354.46	1216.58	0.0402	5.02	17.23
F	2.37	296.06	1154.9	1.68	209.34	816.44	3.06	381.69	1488.8	0.0433	5.41	21.09
G	1.79	223.93	1010.3	1.16	145.48	656.38	2.12	265.27	1196.85	0.03	3.76	16.96
H	2.5	312.17	1274.7	1.77	220.76	901.25	3.22	402.48	1643.4	0.0456	5.70	23.28
I	2.04	256.13	876.49	1.44	181.13	619.4	2.63	330.24	1129.7	0.0373	4.68	16

**Fig. 3:**
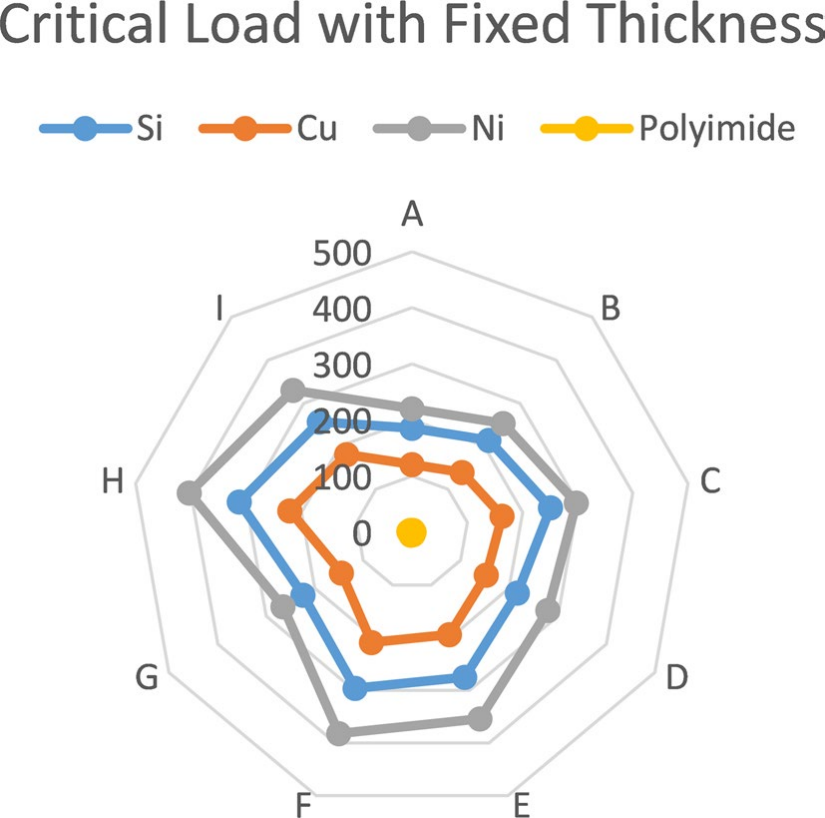
Illustrates the values of critical loads with different designs and materials with fixed thickness of 100 μm

Figure 3 illustrates the values of critical loads with different designs and materials with fixed thickness of 100 μm.

**Fig. 4:**
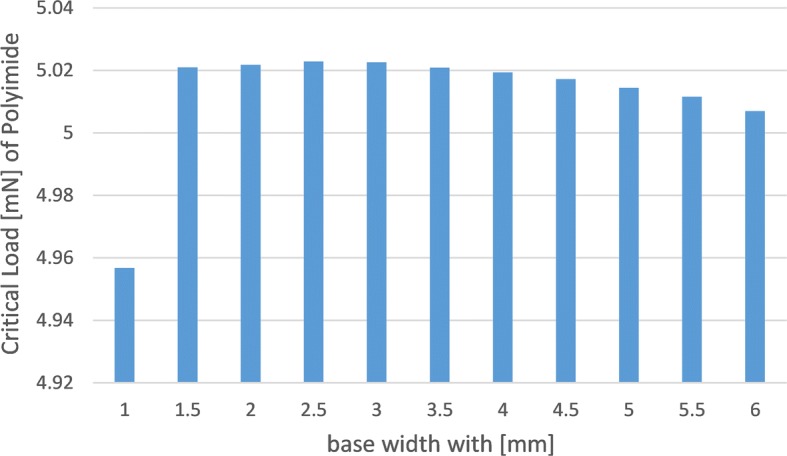
The effect of different base width on the critical load (design E with Polyimide material)

**Fig. 5:**
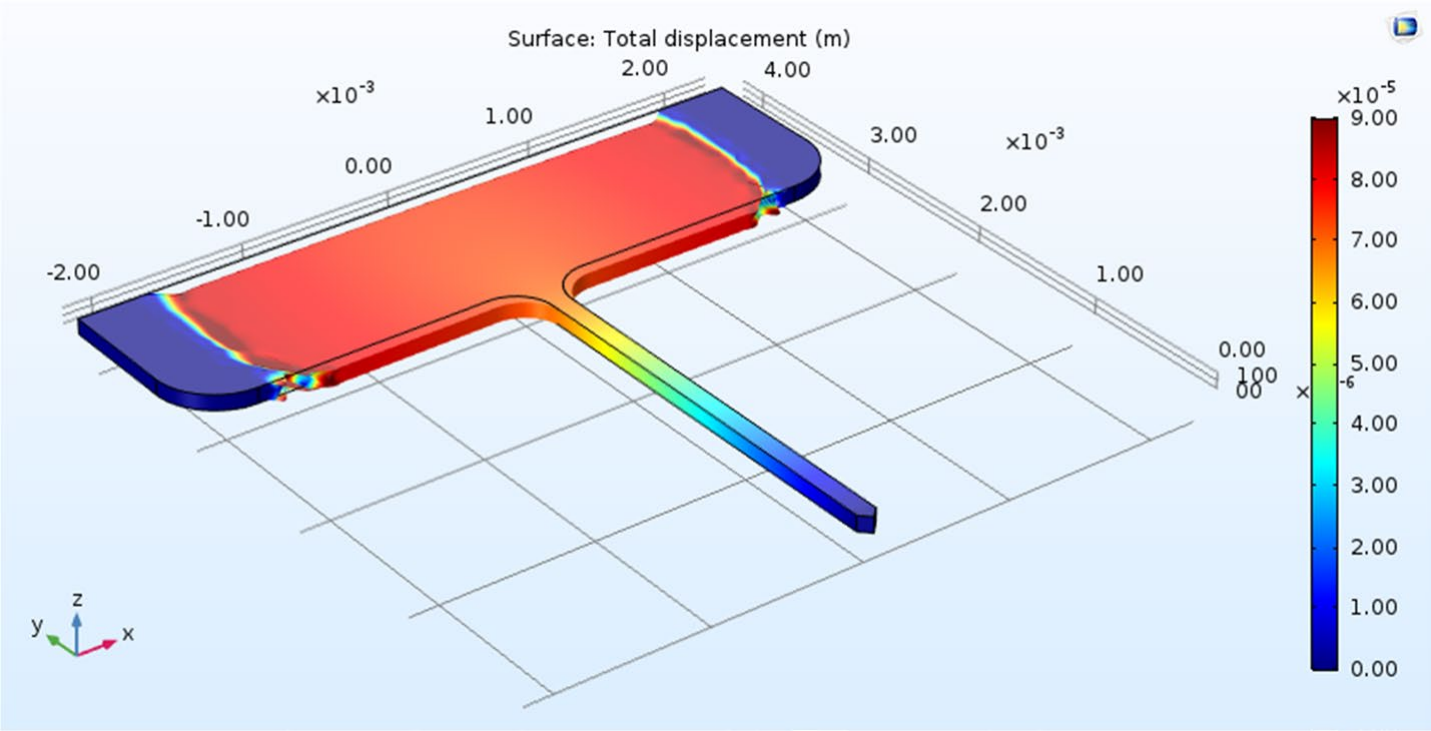
The distortion with very large base (design E with Polyimide material)

Layouts E, F and H have wider bases than A, B and G, but are narrower than C and I, which explains their high resistance to buckling as illustrated in Fig. 4. When the base becomes much wider, the critical load decreases because the shank width is so small compared with the base and produces an unstable design; this is proposed in Fig. 5.

The design with wide base is not suitable for the rats because their brains are very small with a surface area of 6 cm^2^ [15], however, it is suitable for human’s brain. Silicon and Nickel electrodes exhibit superior performance in resisting buckling failure. Nickel electrode with thickness of 30 μm would survive the applied axial force. The modulus of elasticity for Silicon and Nickel are of close values ($E_{silicon}=170\,GPa, E_{Nickel}=220\,GPa$), accordingly; the critical loads for both materials are nearly the same value. On the other hand, Polyimide structures have very low critical loads due to their mediocre module of elasticity, and 30 μm shanks would fail.

### Axial loading

**Fig. 6:**
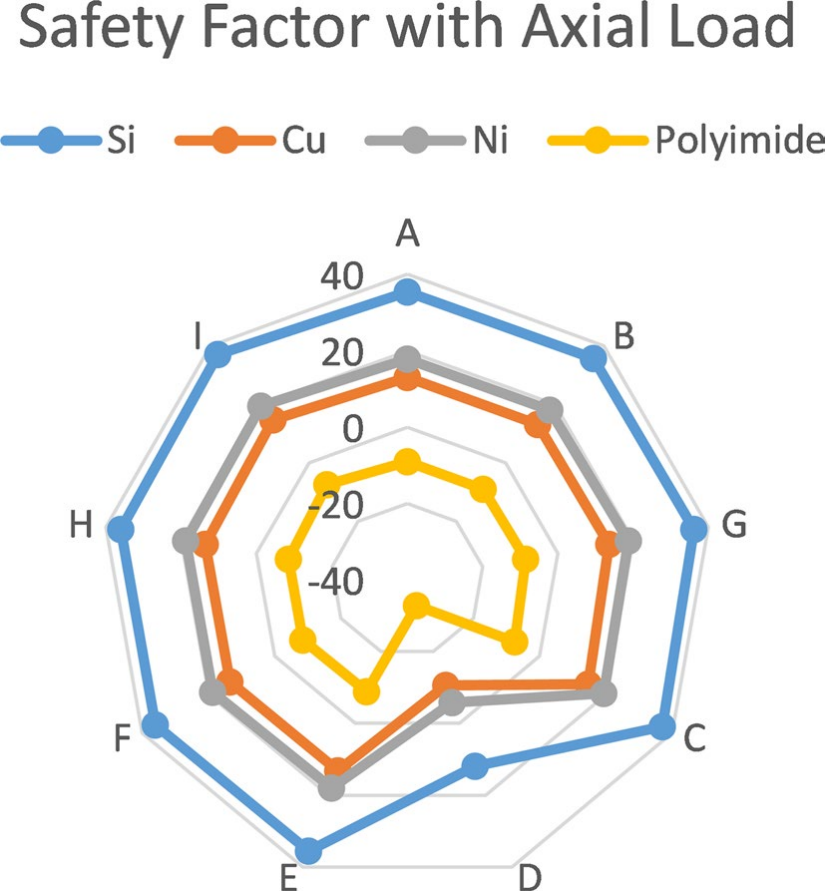
The log diagram for safety factor with axial load the different results of safety factors with axial load for different designs with different materials and fixed thickness

The simulation results illustrate the superiority of Silicon electrodes in resisting axial loading. Silicon shanks with thickness of 50 μm achieve an acceptable safety factor of 20 throughout the critical spots except for layout D. The asymmetry of layout D concentrates the tensile stresses along one of the sides, which cause the safety factor to drop below 5. The asymmetry and sharp edges of layout D have a huge effect on the static simulation when applying axial force to calculate the safety factor, but have no effect on buckling failure. As for the 50 μm Silicon electrodes, several designs show acceptable performance. Nickel electrodes come after the Silicon electrode in failure resistance. Most of the 70 μm Nickel structures have a minimum safety factor of 5 which would survive axial compression and the minimum required thickness is 120 μm to achieve the targeted safety factor of 10. On the other hand, Polyimide has bad mechanical properties compared to Silicon and Nickel, and the minimum thickness required for Polyimide to survive the mechanical loading exceed 200 μm as shown in Fig. 6.

Although Silicon achieves the mechanical performance required, for chronic electrode fabrication flexible substrates are preferred. Flexible substrates have the ability to restore its original shape after the force is eliminated, because it elastically responds to external stresses. Increasing the axial force beyond the yield point would cause permanent deformation without fracture till the ultimate strength is reached. In contrast, brittle (Silicon) electrodes would endure fracture right after crossing the yield point so these are not preferred for long term in vivo applications.

Furthermore, Polyimide is easy to process and is biocompatible; these properties make it eligible for electrode implementation.

### Shear loading

During implantation and operation, lateral forces normal to the shank induce shear stresses, which affect the electrode and create off plane deformation. In this simulation, the electrode is subjected to 1 mN shear force equivalent to the value of the force required for electrode insertion [16]. Fixed-free support conditions are assumed.

The applied shear forces on the electrode shank produce compressive stresses on one side and tensile stresses along the other. Silicon electrodes analysis shows that the side undergoing compressive stresses does not endure failure, as the ultimate tensile strength for the brittle material is lower than the compressive stress. In contrast, Nickel electrodes of different layouts and thicknesses show large safety factors along the compressed side. The results depend on materials to determine the minimum thickness of each layouts to prevent fracture during insertion assuming a single crystal defect free structure. However, layout D with Silicon showed vulnerable response to shear stress at 100 μm.

**Fig. 7:**
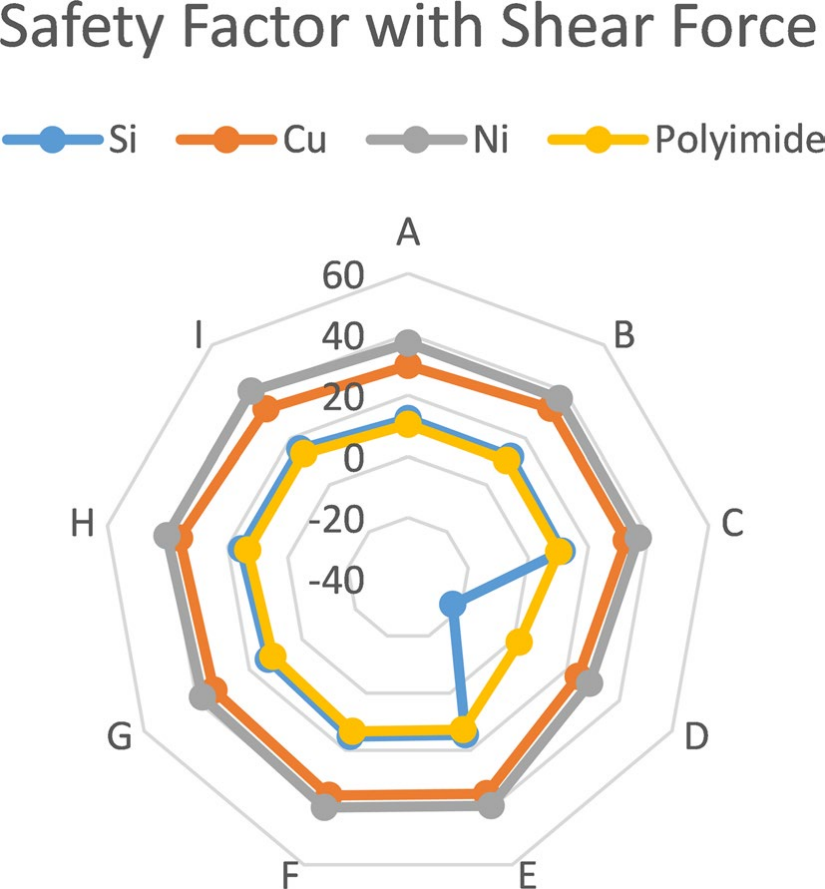
The log diagram for safety factor with shear load illustrates the different results of safety factors with shear load for different designs with different materials and fixed thickness

The safety factor distribution contours for shear loaded with different materials and different layout electrodes are plotted in Fig. 7. Nickel shanks as thin as 40 μm demonstrate acceptable performance with a minimum safety factor of 5. As for Polyimide, they are more vulnerable to shear stresses, and this is rectified by increasing the electrode thickness over 100 μm to realize the required performance.

### Electrode design insights

The previous results illustrate that Nickel exhibits superior resistance to buckling failure during implantation followed by Silicon. Besides that if fabrication costs are taken into account, the Polyimide material has the lowest cost which will cost almost $900. Nickel and Copper come in the second rank with $1800 and then applying the cost of a gold thin film. Using Si as the material costs around $2100.

In this part, a comparative between all designs is carried out to indicate which design is better. The comparative depends on the values of FOM. The best design has the largest value of FOM. The FOM depends on the different values of safety factor for each design that are calculated from mechanical analysis as well as the cross section area of the shaft and the fabrication cost of each material. All the other characteristics are constant for all designs such as impedance and noise because of the pads of all electrodes in these designs are constant.

**Fig. 8:**
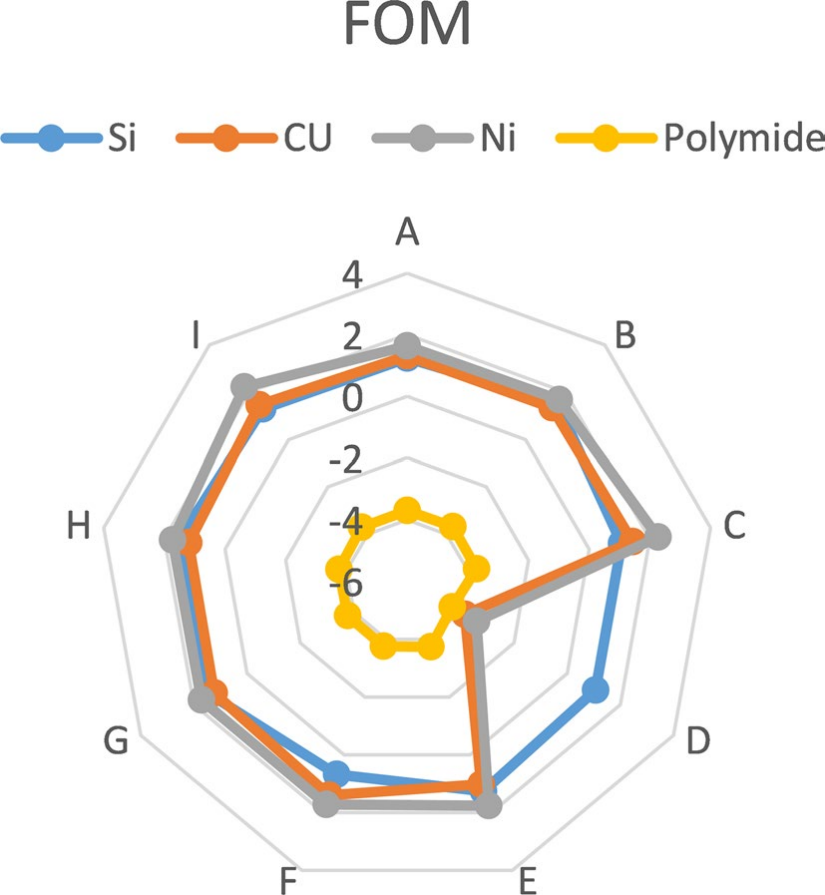
FOM. The FOM for several designs with different materials

Figure 8 illustrates the results of FOM. It shows that the design C and I with Nickel material give the highest value of FOM but its bases are not compatible with rats because they are very large. Design H follows them so it is considered the best design. Design D with Polyimide material is considered the worst design because it gives the worst FOM.

## Discussion and conclusion

This paper presents a review of several prototypes of the proposed electrodes, which are analyzed to satisfy the design requirements. Design H with Nickel material gives the best results which is suitable for rats. Design C and I with Nickel material have the highest FOM but are not compatible with rat brain.

On the other hand, the Polyimide electrodes do not undergo the axial loading which induces fractures and are vulnerable to failure. Shear analysis results are in favor of Nickel electrodes due to its ductile properties, and Polyimide designs with thick cross-sections can replace silicon.
